# Gut immune responses and evolution of the gut microbiome—a hypothesis

**DOI:** 10.1093/discim/kyad025

**Published:** 2023-11-23

**Authors:** Mark Viney, Louise Cheynel

**Affiliations:** Department of Evolution, Ecology & Behaviour, University of Liverpool, Liverpool, UK; Univ Lyon, Université Claude Bernard Lyon 1, CNRS, ENTPE, UMR 5023 LEHNA, Villeurbanne, France

**Keywords:** evolution, microbiome, IgA

## Abstract

The gut microbiome is an assemblage of microbes that have profound effects on their hosts. The composition of the microbiome is affected by bottom-up, among-taxa interactions and by top-down, host effects, which includes the host immune response. While the high-level composition of the microbiome is generally stable over time, component strains and genotypes will constantly be evolving, with both bottom-up and top-down effects acting as selection pressures, driving microbial evolution. Secretory IgA is a major feature of the gut’s adaptive immune response, and a substantial proportion of gut bacteria are coated with IgA, though the effect of this on bacteria is unclear. Here we hypothesize that IgA binding to gut bacteria is a selection pressure that will drive the evolution of IgA-bound bacteria, so that they will have a different evolutionary trajectory than those bacteria not bound by IgA. We know very little about the microbiome of wild animals and even less about their gut immune responses, but it must be a priority to investigate this hypothesis to understand if and how host immune responses contribute to microbiome evolution.

## The gut microbiome

As is now well known, the gut microbiome comprises a very large collection of prokaryotic and eukaryotic cells, though here we will focus on the largest component, the bacteria. The microbiome provides a number of services to its host so it is crucial for host health and wellbeing [1], but some members of the microbiome can also harm the host. The microbiome is a complex mixture, whose composition varies among individuals, can change over time, and can be perturbed by a range of factors.

Most of what we know about the gut microbiome comes from studies of laboratory animals (mainly mice), people, and some livestock species. While there are clear advantages of using laboratory animals, for example when they capture relevant traits of another species (e.g. humans), or of the same species in a different environment (e.g. the wild), there are also problems. Not only do laboratory animals’ microbiomes differ substantially among laboratories and suppliers but also the microbiome can affect other host traits, such as immune function and resistance to infection, giving aberrant results [2, 3]. For example, laboratory mice that would otherwise be killed by an Influenza A virus infection in the lung survive if they have received a gut microbiome transplant from wild mice [4]; lab mice that normally immunologically reject the gut worm *Trichuris* are fully susceptible to it if they live in outdoor enclosures and acquire a wild microbiome [5]. Therefore, there must be some concern that the work of the large international community studying laboratory animals’ microbiomes might be discovering phenomena that are only relevant in the laboratory and missing impacts of the microbiome that occur in wild animals. This, in turn, suggests that to understand animal microbiomes it is necessary (and perhaps ideal) to study the microbiome in wild animals directly.

That said, we know rather little about wild animals’ microbiomes, except that they are usually different than lab-maintained con-specifics, and that they can be more diverse than lab animals’ [6]—e.g. *c.* 30–50% more diverse in wild mice (Shannon diversity index and abundance by phyla [4, 6–11];) (Box 1). Wild animals’ microbiomes are different likely because wild animals differ in a wide range of factors—such as diet, reproductive status, infections, and immune state—that are often heavily constrained in laboratory animals, and these factors affect the composition of the microbiome. In the wild, individual animals will also quantitively vary in these factors, so driving inter-individual variation in their microbiomes. While this among-individual variation in microbiomes might seem unhelpful noise, it is actually the result of different biology of individual hosts, so showing the breadth of factors that act and interact to affect the microbiome.

Box 1. **Measuring microbiome diversity**

***Sequencing*.** DNA sequencing has revealed the complex composition of the microbiome. The most widely-used approach to determine the composition of the microbiome is sequence analysis of fragments of (multi-copy) ribosomal RNA (rRNA) coding genes, 16S for prokaryotes and 18S for eukaryotes. Metagenomic sequencing can also be used, which determines more (or all) of the relevant microbial genome. Bioinformatic analyses of rRNA or metagenomic sequence data can then try to identify the relevant taxon, but this is only possible if the focal taxon has previously been sequenced. Alternatively, the sequence data can be analysed agnostic to taxon identity. A common and attractive approach here is to use amplicon sequence variants (ASV), which are groups of common, near-identical, sequences. Both taxon identification and ASVs can be used in measures of alpha and beta diversity, below.

***Alpha diversity*.** Alpha diversity measures the microbiome of an individual host. It can be measured simply by taxon or ASV richness, which is a direct count of the number of taxa or ASVs. Richness does not account for the relative abundance of different taxa/ASVs, but there are other measures that do, for example, Shannon’s Index and the Chao-1 index. Richness can be increased by the presence of very rare taxa/ASVs (for example a single individual), whereas these other metrics are less sensitive to rare taxa/ASVs.

***Beta diversity***. Beta diversity measures the microbiome as taxa or ASVs at the scale of a group or population of hosts. A commonly used metric is the Bray–Curtis dissimilarity, which calculates the dissimilarity between two populations, with 0 showing that the two populations are identical, and 1 showing that they are completely dissimilar. If we consider a single host’s microbiome as one population, then Bray–Curtis is used for the microbiomes of a group or population of hosts and measures the overall (dis)similarity of the microbiome of this group of hosts.

***UniFrac.*** The diversity of the microbiome can also be measured by the UniFrac statistic. This measures the phylogenetic similarity among the microbiomes that are being compared. In so doing, this is wholly different from Bray–Curtis and other measures of beta diversity, which do not consider the phylogenetic relationship of the taxa in the microbiome.

***Quantification*.** The now standard approach to analysing the composition of the microbiome is to use the abundance of sequences belonging to a taxon or ASV as the measure of its abundance. From this, the composition of the microbiome is then commonly described proportionally; that is, each taxon or ASV is reported as some proportion of the whole microbiome. This approach is therefore unable to account for changes in the absolute abundance of the microbiome, or changes in the abundance of component taxa. For example, compare two individuals differing 2-fold in the number of bacteria of taxon A. The proportionate method would show a greater proportion of A, but also a consequent decrease in the proportional abundance of other taxa. However, a fully quantitative method would show that taxon A was twice as abundant, while, for example, all other taxa maintained the same abundance, therefore giving very different measures of microbiome composition than the proportionate approach. Where studied, the microbial load of individuals’ microbiomes has been found to vary, and so too has abundance of different microbial taxa [83].

## Controlling the composition of the microbiome—top-down and bottom-up processes

Clearly the composition of the microbiome is important—the human microbiome is generally stable over time and deviation from this can cause harm to the host [12]. Therefore, understanding the processes that control the composition of the microbiome is of interest and importance. However, in beginning to think about this, it is very important that we are clear about what is meant by composition of the microbiome. The microbiome composition can be measured, and so defined, in different ways, which can be (i) simply the species composition, (ii) other abundance-controlled measures, or (iii) bacterial phylogenetic diversity (Box 1). Thus, when thinking about the control of the composition of the microbiome we need to be clear about what aspect or measure of the composition is being considered.

A useful way to approach thinking about the composition of the microbiome is to recognize that it is an ecosystem, where direct and indirect interactions between bacterial taxa in the microbiome affect the presence and abundance of bacterial taxa. For example, the human preterm-infant microbiome is assembled in a predictable pattern, with much of this driven by among-species interactions [13, 14]. Thus, while species are acquired from the environment somewhat haphazardly, their establishment and subsequent growth within the microbiome depends on the presence of other species, which therefore generates a certain predictability in the composition of the assembled microbiome [15]. Experimental introduction of microbial taxa into mice also shows that order-of-arrival in the gut affects colonization success [16]. These among-species interactions are, in the language of ecology, bottom-up processes. While these among-species interactions can be seen, for example, in the establishment of the preterm-infant microbiome, these processes are actually occurring all the time within microbiomes and so contribute to determining the composition and structure of the microbiome.

It is self-evident that the gut microbiome exists within its host and so host physiology and immune state create (and change) the environment in which the microbiome exists. In this way, the host can alter the composition of the microbiome. Again, in the language of ecology, the microbiome is therefore affected by top-down host factors. For example, a seasonal change in a wild animal’s diet could lead to a change in the species composition of the microbiome because the different food present in the gut will favour some bacterial species and disfavour others. Also, one can envisage that reproductive hormonal changes in female animals could result in a change in gut physiology thereby also altering the species composition of the microbiome. There are also host genetic effects on the microbiome, which are another manifestation of top-down processes. In wild mice (*Mus musculus domesticus*) the genetic distance among individual mice predicted the composition of their microbiome, and these effects persisted in animals when they were in a common laboratory environment [11]. These effects also can be seen by analysis of the heritability (the proportion of the among-individual variation in microbiome composition that is attributable to host genetics) of the microbiome composition. In humans, one study found that the abundance of 5% of taxa in the microbiome was heritable, whereas in a second study it was of 37% of taxa [17, 18]. The composition of the baboon gut microbiome has also been found to be heritable, though generally with low values, though the heritability changed over seasons, etc. pointing to the effects of the wider environment on the microbiome [19]. In lab mice, some 20 quantitative trait loci have been found that affect the composition of the microbiome [20]. But any top-down change in the composition of the microbiome will also have consequences for the bottom-up, among-species interactions in the microbiome [21].

In summary, both bottom-up and top-down processes affect the composition of the microbiome and, presumably, its function and so its contribution to host health, wellbeing, and evolutionary fitness. Therefore, understanding how the composition of the microbiome is determined is necessary to understand the normal biology of animals.

## Bacterial evolution

It is axiomatic that bacteria in the microbiome will evolve, as all organisms do. In thinking about this, there are at least two conceptions of microbiome evolution. One is that microbiome evolution is a quantitative change in the composition of the microbiome, that is a change in its alpha diversity (Box 1). This could occur because of both bottom-up processes (as hosts acquire new bacteria and then there are different among-species interactions) and/ or top-down processes, due to changes in the host diet, physiology, immune state, etc [21–23]. A second conception of microbiome evolution is the molecular evolution of bacterial taxa within the microbiome [21–23]. In thinking about this we need to focus on genotypes (or strains [24]) of bacteria, recognizing that it is these (rather than species) that are competing with each other and that are subject to natural selection. As the evolution of genotypes occurs the bacterial species will still exist and persist in the microbiome, but the species’ component genotypes will have changed.

Different bacterial genotypes will evolve by processes of mutation and horizontal gene transfer [25], which will occur on short time scales, and so within the lifespan of the host. The potential for evolution is huge; for mutations only, given the large population size of many bacteria in the microbiome, their relatively small genomes, and assuming a conservative mutation rate then every position in the genome could be theoretically be mutated in every bacterial generation [24].

There has been some study of evolution of bacteria within the microbiome. In humans, over timescales of multiple months there is evolutionary change of resident bacterial strains (with the acquisition of new strains occurring only rarely) [22]. A 6 year-long study of laboratory mice colonized with 12 bacterial taxa, showed that the overall composition of this simple microbiome was stable, but that there was evolution of within-taxa strains that then co-existed, though a change in host diet brought about a rapid shift in the microbiome’s composition [26]. As *Escherichia coli* adapted to the gut of laboratory mice, soft genetic sweeps—the multiple occurrences of beneficial mutations—occurred in the *E. coli* population, with inter-genotype competition, but this bacterial evolution differed in young and old mice, with patterns suggesting that the old mouse gut was a comparatively more harsh environment for *E. coli* [27, 28]. In a longitudinal study of a single human microbiome, focusing on 36 bacterial species, there were different levels of genetic variation within each species that changed over time with this likely being driven by selection [29]. Though in contrast, longitudinal study of a single clone of *E. coli* in a single human microbiome found limited genetic diversity and no evidence of selection [30]. Analysis of some bacterial species in the gut microbiome of people from 14 different (urban *vs.* rural) populations showed that horizontal gene transfer was very common and frequent and that this occurred more in individuals from urban settings [31]. Within the human microbiome, *Bacteroides fragilis* was found to be genetically diverse, with this diversity having arisen within individuals, with horizontal gene transfer selection having occurred [32]. Analysis of the genomic evolution of 42 bacterial pan genomes found that genes coding for secreted products evolved faster than other genes [33]. These secreted proteins are, presumably, how bacteria interact with each other but also how they interact with their host, and so evidence of their accelerated evolution is particularly relevant to bacteria in the gut microbiome.

In summary, there is good evidence that bacteria in the microbiome are genetically diverse, that this diversity can change and that selection can act, changing the bacterial genotypes comprising the bacterial species, therefore changing the composition of the microbiome.

## The gut immune response and IgA

The gut has a fully functioning immune system that, together with other host physiological processes, maintains gut integrity in the face of the large microbial population resident in the gut. The core of this gut immune system is the gut-associated lymphoid tissue, particularly Peyer’s patches (PP), which occur along the length of the gut, and mesenteric lymph nodes. Epithelial microfold cells and dendritic cells sample antigens of microbes that are associated with, or attempting to penetrate, the intestinal epithelium, resulting in induction of T and B cells in the PPs [34, 35]. T-cell responses remain confined to the intestinal tissue, whereas B cells produce IgA that is secreted across the intestinal epithelium. Secreted IgA is a dimer, covalently bound to a secretory component [34]. Therefore, within the gut lumen itself, the only adaptive immune response is secreted IgA. The gut microbiome is necessary for the production of IgA, as shown by its near absence in germ-free animals [36–38].

IgA is abundant, with 80% of all human plasma cells secreting IgA, with the gut containing *c.* 70% of all antibody-producing plasma cells [39, 40], and humans secrete *c.* 5 g of IgA into the gut daily [41]. Normally other antibody isotypes are not present in the gut, though this can occur when there is gut pathology (e.g. gut IgG in Chron’s disease, ulcerative colitis; inflammatory bowel disease [42]) or partly replacing IgA in settings of IgA deficiency (which 0.2% of the Caucasian population are, which is not lethal, though individuals can have a range of symptoms) [43]. However, whether or not IgA deficiency occurs in wild animal populations (and if so with what prevalence) is not known.

When an antibody response develops, immunoglobulin genes can undergo somatic hypermutation (SHM), which increases the affinity of the antibody for the target antigen—this is the standard view of how a circulating immune response develops. However, lab mouse studies suggest that not all the gut IgA response appears to be affinity matured in this way, and so rather the gut IgA response consists of both high-affinity (sometimes called ‘induced’) IgA (resulting from SHM) and poly-reactive (sometimes called ‘natural’) IgA responses (not resulting from SHM processes) [44, 45]. Therefore, lab mouse IgA responses are generated in a T-cell independent, some in a T-cell dependent, fashion though the significance of these observations continues to be discussed [44]. However, in humans (which perhaps are immunologically closer to wild animals) then there is clear evidence of SHM in intestinal IgA responses so that these responses are T-cell dependent [46, 47].

IgA, as other antibodies, can bind antigen through its Fab regions, but IgA also has non-canonical binding, *via* parts of the antibody molecule other than the Fab regions. For IgA, much of the non-canonical binding occurs *via* the extensive glycosylation of the IgA molecule [44]. This glycosylation makes IgA protease-resistant, so prolonging its half-life in the gut. Further, this non-canonical binding might be important in IgA’s interaction with bacteria, in two ways. First, the surface of many bacteria is glycosylated, which might therefore be important in IgA’s binding to bacteria. Second, these glycan-based interactions might allow IgA molecules to recognize multiple bacterial species. Specifically, it has been found that IgA monoclonal antibodies can recognize multiple bacterial species (though perhaps most plasma cells produce antigen-specific IgA) [44, 48, 49]. This phenomenon may be because of interactions between glycans on the antibody molecule and bacterial glycans, and/or that antibodies recognize glycan epitopes, and/ or that there is cross-reactivity between non-glycan epitopes.

But, all of this work, and so our understanding of gut immune responses in general, and IgA responses in particular, has come from work in laboratory animals and people. Nothing, as far as we are aware, is known about wild animals’ gut immune responses. In wild mice, *Mus musculus domesticus*, faecal IgA concentrations have been measured. The faecal IgA concentration of wild mice varied extensively (2—1178 μg/ g faeces), with the very high concentrations most likely in older animals [50], this variation was more so than in lab mouse controls (130–380 μg/ g faeces), though overall there was no difference between mice from these two sources [50]. Pet shop-acquired mice have higher serum IgA concentrations than lab mice [51].

Overall, the IgA response appears to corral the microbiome to the gut and can alter its composition [1, 36, 45, 52–54]. For example, in antibody-deficient mice the gut microbiome has a different composition compared with control animals [54]; though in contrast, the gut microbiome maintained a similar composition in IgA-replete and IgA-deficient people, suggesting a more limited role of IgA in moulding the composition of the microbiome [43]. Here though, it is important to be clear about what measures of microbiome composition (Box 1) are being considered. Comparing the evolution of *E. coli* strains in immunologically normal and immune deficient (*Rag2*^*−−*^, lymphocyte deficient) mice showed that these different immune environments (interacting with their microbiomes) provided different selective environments for *E. coli* [55]. The microbiome of a range of immune-deficient mice (*Rag1*^*−/−*^, B and T-cell deficient; *lghm*^*−/−*^, B-cell deficient; *Cde3*^*−/−*^, T-cell deficient) had a lower species richness compared with immunologically normal controls [54]. Laboratory mice either unable to class switch to produce IgA nor undergo SHM (AID^*−*/*−*^), or mainly SHM deficient (AID^G23S^), had a severely altered microbiome (compared with normal controls) through reconstitution of normal IgA function restored microbiome composition [56, 57]. In these studies using immunodeficient animals where the microbiome is altered compared with non-immunodeficient animals, then these effects may be IgA-dependent but may also be due to other, wider effects of the immunodeficiency on the animal’s physiology. IgA clearly interacts with bacteria in the microbiome, because many bacteria are coated with it [39, 58–61]. Flow cytometry has been used to separate bacteria that are bound by IgA, and those that are not, henceforth IgA^+^ and IgA^-^ bacteria, respectively. The UniFrac distance (Box 1) of IgA^+^ and IgA^*−*^ bacteria from laboratory mice differed, though this difference was reduced or removed in *Cde3*^*−/−*^ (T-cell deficient) mice [62].

**Figure 1.**
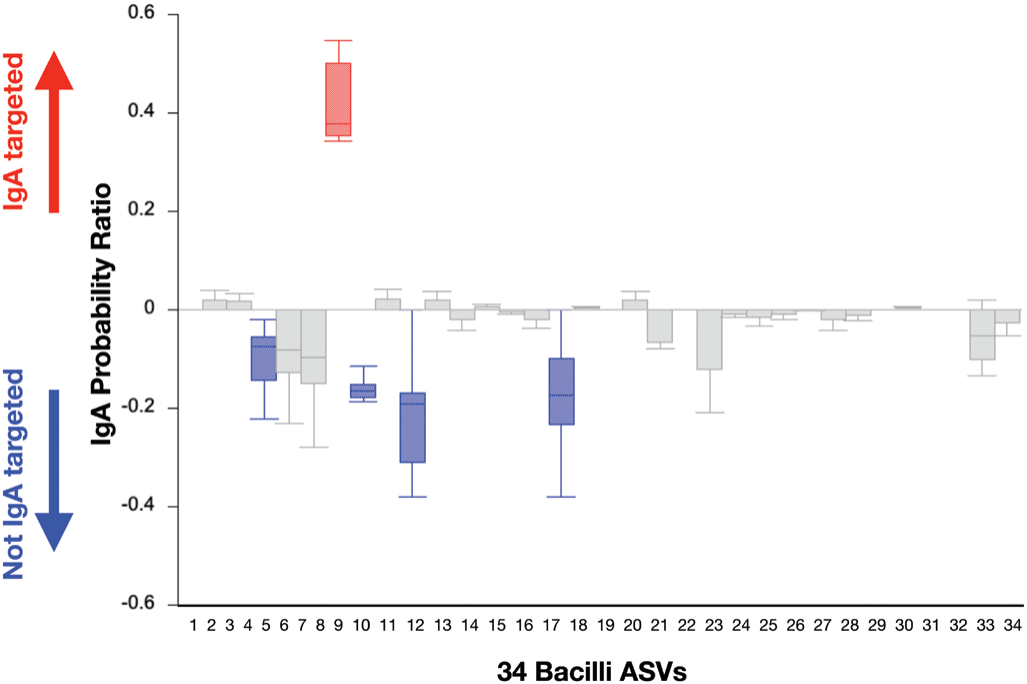
IgA targeting of Bacilli ASVs. Faecal bacteria from six wild mice (*Mus musculus domesticus*) were prepared, stained with anti-IgA and FACS sorted into IgA^+^ and IgA^−^ fractions, which were then 16S sequenced, assigned to ASVs, and IgA probability ratios (after [59]) calculated for 34 ASVs. For the five highlighted ASVs the first to third quartile values are above (red) or below (blue) zero. Whiskers show minimum and maximum probability ratio values. Detailed methods are in Supplementary Information.

Flow cytometry-based separation of IgA^+^ and IgA^*−*^ bacteria, followed by DNA sequencing [58] has been used to understand what bacteria are in each fraction, and whether IgA binding is directed to pathogenic (or potentially pathogenic [2]) bacteria. The results of this have shown that many (perhaps most) of the IgA^+^ bacteria are commensal, though some appear to be involved in gut pathology [58, 60]. In our own studies of the faecal microbiome of wild mice, we have flow cytometry-separated and 16S-sequenced IgA^+^ and IgA^*−*^ bacteria (Figure 1). For 34 bacilli ASVs there was one enriched in the IgA^+^ fraction, four enriched in the IgA^*−*^ fraction, with the remaining 29 not enriched in either way. One interesting question that needs to be addressed is the extent to which the same bacterial genotypes or ASVs are in IgA^+^ or IgA^*−*^ fractions among different hosts and across different populations of hosts. The extent of the inter-individual host variation of gut IgA responses and how these are directed or not to bacterial genotypes, especially in wild animals, is a key unknown.

The functional effects of IgA’s binding to bacteria is not yet fully known [1, 63–65] but it has been shown to clump bacteria [44, 63], inhibit bacterial cell division [66], provide an altered within-gut niche for the bacteria [34, 67, 68], and change bacterial gene expression and metabolism, including of bacterial-derived toxins [34, 44, 63, 67–70].

In summary, the bacterial microbiome is necessary to generate what becomes a large gut IgA response that, overall, keeps the microbiome in the gut, with IgA able to affect the composition of the microbiome. IgA directly binds to a proportion of the bacterial cells in the microbiome, with this IgA binding potentially affecting the IgA-bound bacteria.

## What is the effect of the IgA response?

Or to put this question in a more biologically rigorous form, what is the evolutionary fitness advantage of making a gut IgA response? Immune responses are energetically expensive to make and so animals will only invest in them if they bring benefit [20]. Therefore, we can surmise that gut IgA responses are being made because they do something useful, important and beneficial to the host, which could include controlling the microbiome’s composition to the host’s benefit. Presumably, the gut host immune response seeks to maintain gut integrity, to attack and neutralize pathogens, but while maintaining the ability to absorb nutrition from the gut. Clearly, the IgA and associated gut cellular response do protect the integrity of the gut and keep the bacterial microbiome in the gut, where it belongs, but this must be a balance of having a sufficiently highly activated immune response to stop or ameliorate the harm that pathogens can cause, but not one that interferes with nutrition. But beyond this, generating an IgA response that favours bacteria that are beneficial to the host and disfavouring bacteria that are non-beneficial (or even harmful), must maximize host fitness. With this perspective, is the binding of IgA to a proportion of the bacterial cells in the microbiome the attempt of hosts to choose between different bacteria to create a microbiome that maximizes its contribution to host fitness?

But of course, the bacteria in the microbiome are not passive partners, because they are also evolving and seeking to maximize their fitness. This leads to the inevitable question, what is the evolutionary effect of the gut IgA response on gut bacteria? We propose the hypothesis that IgA’s binding to bacteria acts as a selective force so that IgA^+^ bacteria have an altered fitness compared with IgA^*−*^ bacteria. With IgA acting as a selective force, we predict that the IgA^+^ bacteria will evolve molecularly faster, or at least differently, compared with IgA^*−*^ bacteria. For this idea to be correct the binding of IgA to bacteria needs to alter bacterial fitness in some way, compared with bacteria not bound by IgA. Because bacteria have a very short generation time compared with their hosts, this IgA-driven evolution would occur during the host’s life. Indeed, there is the possibility that some of the already-observed evolution of bacteria in the microbiome (Section “The gut immune response and IgA”, above) may be the result of IgA responses against bacteria in the microbiome.

In conceptual support of our hypothesis (and indeed its inspiration) are the multiple examples of host immune responses imposing selection pressures on pathogens, driving their molecular evolution. Well studied examples include: *Streptococcus pneumoniae, Neisseria meningitidis, Staphylococcus aureus, Plasmodium falciparum* (the malaria parasite), Influenza A, Dengue, HIV, Hepatitis C, Foot and Mouth Disease Virus, and *Mycoplasma gallisepticum* [33, 71–80]. In these systems, host immune responses kill or constrain certain antigenic types (i.e. strains [81]) that hosts have previously encountered, so facilitating the emergence of new strains. Immune responses therefore drive changes in these populations of pathogens. This immune-mediated selection can be detected by phylogenetic analysis of strains across a host population—the common process driving strain divergence is immune-dependent selection, but the infection biology of each pathogen (prevalence, infectivity, persistence, etc.) modifies these phylogenetic relationships [73]. In our hypothesis, the gut IgA response directed at bacteria within the microbiome is the evolutionary selective force and driving the microbiome’s evolution.

While IgA responses are directed to gut pathogens it appears that many commensal bacteria are also bound by IgA, and so our hypothesis is that there will be IgA-driven evolution of commensal bacteria within the microbiome.

## Testing the hypothesis of IgA-driven evolution of gut bacteria

There are a number of approaches that can be used to investigate this hypothesis. The most obvious approach is to longitudinally sample the microbiome of hosts, then separate and metagenomically sequence the IgA^+^ and IgA^-^ fractions and measure the molecular evolution of genotypes across time. This would be a direct test of the hypothesis that bacteria in the IgA^+^ fraction have a different molecular evolutionary trajectory than those in the IgA^-^ fraction. This could be done using laboratory animals, though we would favour doing this in wild populations, recognizing the different immune responses of wild animals compared with those of laboratory animals [50, 82]. But there are other approaches that could be used. One approach would be to transplant artificially marked bacterial strains into animals and to track the evolution of those strains while also measuring the IgA and other immune responses directed towards them. This approach could involve different bacterial taxa and include mixtures of differently marked strains. Also, the IgA dependency of any observed effects could be tested by using immune-manipulated and knock-out mice, potentially transplanted with wild mouse microbiomes [4].

## Significance of IgA-driven evolution of the bacterial microbiome

If our hypothesis is correct and IgA^+^ bacteria have an altered evolutionary trajectory compared with IgA^-^ bacteria, this process will drive changes to the genotypes comprising bacteria species, and so change the composition of the microbiome. Because individual animals will make their own individual immune responses, the IgA selection pressure will probably differ among individual hosts, so that the same bacterial species may be evolving differently among individual hosts. Such inter-individual effects are likely to be more obvious and important in wild animals, rather than in laboratory animals.

Because of the importance of the microbiome in health and well-being, there is considerable interest in manipulating the microbiome of people and animals for therapeutic purposes. The principal approach to this is to change the composition of the microbiome by adding new bacteria (by ingestion of bacterial mixes or by microbiome transplant) or by the use of antibiotics. IgA-driven bacterial evolution could lead to the failure of attempts to manipulate the composition of the microbiome. That is, one could introduce a bacterial strain of therapeutic interest, but its subsequent within-host evolution could result in the loss of the anticipated therapeutic benefit. Equally, but in contrast, immune-driven evolution of the microbiome could potentially be used therapeutically. That is, there is the potential to immune-drive the evolution of bacterial genotypes for host therapeutic benefit.

## Summary and conclusion

The gut bacteria microbiome is important for animals’ normal health. While the microbiome has been extensively studied in people, livestock, and laboratory animals, there has been very limited study of it in wild animals. The composition of the microbiome can be affected by both bottom-up and top-down processes, and the bacterial genotypes that comprise bacterial species can evolve, with this evolution itself contributing to changes in the microbiome. The gut immune response, particularly the gut IgA response, affects the gut microbiome, with IgA binding to a proportion of bacterial cells in the microbiome. There are a number of potential effects of this binding on the bacteria, but here we hypothesize that this IgA binding will act as selective pressure on IgA-bound bacteria so driving their molecular evolution, thus contributing to changes in the composition of the microbiome. Although much remains to be investigated about immune responses and the microbiome of wild animals, there will likely be considerable inter-individual variation in wild animals’ IgA responses such that any IgA-based selection that bacteria face will vary across individual hosts. It should be of considerable interest to investigate the role of gut IgA responses in driving the evolution of wild animals’ microbiomes and to understand how this contributes to the biology of wild animals.

## Supplementary Material

kyad025_suppl_Supplementary_Data

## Data Availability

Not applicable.

## Abbreviations

ASV: amplicon sequence variants
PP: Peyer’s patches
rRNA: ribosomal RNA
SHM: somatic hypermutation
