# Supplementary material for: Gut immune responses and evolution of the gut microbiome—a hypothesis
Source: Discov Immunol. 2023 Nov 23;2(1):kyad025. doi: 10.1093/discim/kyad025 (PMC10917216; doi:10.1093/discim/kyad025)
Supplement: kyad025_suppl_Supplementary_Data [file kyad025_suppl_Supplementary_Data.docx]

**Supplementary Information**

**Methods for data shown in Figure 1**

**Mice and Faecal Samples**

The study used two female and four male house mice (*Mus musculus domesticus*) that were live trapped in September 2019 on a farm in northern England, UK (location 52°54'36.5"N 1°05'33.4"W), then moved to a conventional animal house where faecal samples were collected from each mouse within 24 hours, and the samples then stored at -80 °C before processing. This work was approved by the University of Liverpool Animal Welfare and Ethical Review Body.

**Sample preparation**

Faecal pellets were incubated in 1 mL Phosphate Buffered Saline (PBS) per 100 mg faecal material on ice for 1 hr and faecal pellets mechanically homogenized with a stirrer and then centrifuged (50 x g, 15 min, 4°C) to remove larger particles. Faecal bacteria in the supernatant were removed (100 μL per sample), washed with 1 mL PBS containing 1% (w/v) Bovine Serum Albumin and centrifuged (8,000 x g, 5 min, 4°C) before resuspension in 1 mL staining buffer. A 20 μL sample of this bacterial suspension was saved as the pre-sort sample for later 16S sequence analysis. For each sample, a FACS negative control was prepared, which consisted of 100 μL of the suspended cellular suspension, added to 400 μL fresh staining buffer. After Vanderputte *et al*. 2017 the remaining bacterial suspension was first stained with 1 μL SYBR Green I (1:100 dilution in dimethylsulphoxide; shaded 20 min incubation at 37 °C; 10,000 concentrate, Thermo Fisher Scientific), and after an additional wash, bacterial pellets were resuspended in 100 μL blocking buffer (staining buffer containing 20 % (v/v) normal rat serum) and incubated for 20 min on ice. Finally, after Palm *et al*. 2014 the bacteria were stained with 100 μL staining buffer containing PE-conjugated anti-mouse IgA (1:12.5; eBioscience clone mA-6E1) for 30 min on ice. Samples were then washed 3 times with 1 mL staining buffer before flow cytometry sorting.

**Flow cytometry**

The bacterial samples were sorted by flow cytometry using a BD FACSAria^TM^ III flow cytometer. Fluorescence intensity was collected at 530 ± 30 nm, together with side (SSC) and forward (FSC) scatter data. Data were processed using FACS Diva software and electronic gating was used to identify events that gave positive signals for each stain (see Sample Preparation). Measurements were performed at a pre-set flowrate of 5,000-10,000 cells / second, using a 100 μm nozzle. Density plots of SSC and green fluorescence generated from the SYBR GREEN stain identified events that were likely cells (*i.e.* bacteria). These selected cells were then secondarily gated using density plots showing SSC and fluorescence generated from the IgA Monoclonal Antibody (mA-6E1, see Sample Preparation) PE stain, thus sorting IgA coated (IgA^+^) and IgA not-coated (IgA^-^) bacteria. In setting the gating strategy we used the following controls: (i) two faecal samples from IgMi mice that have no secreted antibody (Waisman *et al*. 2007, 2008), and (ii) and negative, no-stain, controls (see Sample Preparation) prepared for each mouse that were used as staining and gating controls.

**DNA preparation**

Bacterial samples (both pre-sort and the IgA^+^ and IgA^-^ samples) were suspended in 400 μL staining buffer before adding 250 μL 0.1 mm zirconia/silica beads (Biospec), 300 μL Lysis buffer (200 mM NaCl, 200 mM Tris, 20 mM EDTA, pH 8), 200 μL 20 % (w/v) SDS and 500 μL phenol : chloroform : isoamylalcohol (25:24:1, pH 7.9; Sigma). Samples were chilled on ice for 4 min and then homogenized by bead beating (2 min bead beating, 2 min on ice, 2 min bead beating). After centrifugation (6000 x *g*, 4°C) the aqueous phase was transferred to a Phase Lock Gel tube (Light; 5 PRIME), an equal volume of phenol : chloroform : isoamylalcohol added, and samples mixed by inversion and then centrifuged (16,100 x *g*, 3 min, room temperature). The DNA was then precipitated and resuspended in 50 μL TE buffer (pH 7).

**Sequencing**

The V4 region of 16S ribosomal RNA was then PCR amplified (2-step PCR amplification using 2 μL of gDNA samples per reaction; KAPA Hi-Fi 2x Master Mix; 35 cycles; primer pair F515/R806 (Caporaso *et al*. 2011)). A blank extraction was used as a negative control and a mock community as a positive control (ZymoBIOMICS^TM^ D6305). Sequencing was then performed on an Illumina MiSeq, v2 run (Paired-end, 2x250 bp sequencing, generating an estimated 12 million clusters per run) with additional PhiX to increase the library diversity.

**Data analysis**

Paired-end reads were merged and the data were quality filtered and denoised using the dada2 package on R (v1.16.0; Callahan *et al*., 2016). Both forward and reverse reads were truncated at 240 bp to account for a decrease in sequencing quality. The **amplicon sequence variants (**ASVs) produced were then assigned to taxa using the Silva reference database (Silva version 138.1; Quast *et al*. 2013). Finally IgA^+^ probability ratio scores were calculated for each ASV after Jackson *et al*. 2021.

**References for Supplementary Information**

Callahan BJ, McMurdie PJ, Rosen MJ, Han AW, Johnson AJ, Holmes SP. DADA2: High-resolution sample inference from Illumina amplicon data. *Nat Methods* 2016 13:581-3. doi: 10.1038/nmeth.3869.

Caporaso JG, Lauber CL, Walters WA, Berg-Lyons D, Lozupone CA, Turnbaugh PJ, et al. Global patterns of 16S rRNA diversity at a depth of millions of sequences per sample. *Proc Natl Acad Sci USA* 2011 108:4516-4522. doi: 10.1073/pnas.1000080107.

Jackson MA, Pearson C, Ilott NE, Huus KE, Hegazy AN, Webber J, et al. Accurate identification and quantification of commensal microbiota bound by host immunoglobulins. *Microbiome* 2021 9:33. doi: 10.1186/s40168-020-00992-w.

Palm NW, de Zoete MR, Cullen TW, Barry NA, Stefanowski J, Hao L, et al. Immunoglobulin A coating identifies colitogenic bacteria in inflammatory bowel disease. *Cell* 2014 158:1000-1010. doi: 10.1016/j.cell.2014.08.006.

Quast C, Pruesse E, Yilmaz P, Gerken J, Schweer T, Yarza P, et al. The SILVA ribosomal RNA gene database project: improved data processing and web-based tools. *Nucleic Acids Res* 2013 41:D590-6. doi: 10.1093/nar/gks1219.

R Core Team (2021). R: A language and environment for statistical computing. R Foundation for Statistical Computing, Vienna, Austria. URL https://www.R-project.org/.

Vandeputte D, Kathagen G, D'hoe K, Vieira-Silva S, Valles-Colomer M, Sabino J, et al. Quantitative microbiome profiling links gut community variation to microbial load. *Nature* 2017 551:507-511. doi: 10.1038/nature24460.

Waisman A, Kraus M, Seagal J, Ghosh S, Melamed D, Song J, et al. IgG1 B cell receptor signaling is inhibited by CD22 and promotes the development of B cells whose survival is less dependent on Ig alpha/beta. *J Exp Med* 2007 204:747-758. doi: 10.1084/jem.20062024.

Waisman A, Croxford AL, Demircik F. New tools to study the role of B cells in cytomegalovirus infections. *Med Microbiol Immunol* 2008 197:145-149. doi: 10.1007/s00430-008-0088-z.
